# Fluid Shear Stress Upregulates E-Tmod41 via miR-23b-3p and Contributes to F-Actin Cytoskeleton Remodeling during Erythropoiesis

**DOI:** 10.1371/journal.pone.0136607

**Published:** 2015-08-26

**Authors:** Weiyun Mu, Xifu Wang, Xiaolan Zhang, Sida Zhu, Dagong Sun, Weibo Ka, Lanping Amy Sung, Weijuan Yao

**Affiliations:** 1 Hemorheology Center, Department of Physiology and Pathophysiology, School of Basic Medical Sciences, Peking University Health Science Center, Beijing, 100191, China; 2 Department of Emergency, Beijing Anzhen hospital, Capital Medical University, Beijing, 100029, China; 3 Institute of Microbiology, Chinese Academy of Sciences, Beijing, 100101, China; 4 Department of Bioengineering, University of California, San Diego, La Jolla, CA 92093, United States of America; The University of Hong Kong, CHINA

## Abstract

The membrane skeleton of mature erythrocyte is formed during erythroid differentiation. Fluid shear stress is one of the main factors that promote embryonic hematopoiesis, however, its effects on erythroid differentiation and cytoskeleton remodeling are unclear. Erythrocyte tropomodulin of 41 kDa (E-Tmod41) caps the pointed end of actin filament (F-actin) and is critical for the formation of hexagonal topology of erythrocyte membrane skeleton. Our study focused on the regulation of E-Tmod41 and its role in F-actin cytoskeleton remodeling during erythroid differentiation induced by fluid shear stress. Mouse erythroleukemia (MEL) cells and embryonic erythroblasts were subjected to fluid shear stress (5 dyn/cm^2^) and erythroid differentiation was induced in both cells. F-actin content and E-Tmod41 expression were significantly increased in MEL cells after shearing. E-Tmod41 overexpression resulted in a significant increase in F-actin content, while the knockdown of E-Tmod41 generated the opposite result. An E-Tmod 3’UTR targeting miRNA, miR-23b-3p, was found suppressed by shear stress. When miR-23b-3p level was overexpressed / inhibited, both E-Tmod41 protein level and F-actin content were reduced / augmented. Furthermore, among the two alternative promoters of *E-Tmod*, P_E0_ (upstream of exon 0), which mainly drives the expression of E-Tmod41, was found activated by shear stress. In conclusion, our results suggest that fluid shear stress could induce erythroid differentiation and F-actin cytoskeleton remodeling. It upregulates E-Tmod41 expression through miR-23b-3p suppression and P_E0_ promoter activation, which, in turn, contributes to F-actin cytoskeleton remodeling.

## Introduction

The membrane skeleton is the basis of erythrocyte morphology and deformability. It is the hexagonal lattice structure formed by 6 spectrin tetramers connecting to the short actin filaments at the junctional complex. The membrane skeleton is anchored to the lipid bilayer via ankyrin and band 4.1. The short actin filaments or protofilaments in the junctional complexes have the constant length of ~35–37 nm, which plays important roles in keeping the hexagonal structure and the mechanical property of the membrane skeleton. The short actin filament consists of 6 pairs of actin monomers and is coated by tropomyosins (TMs), with erythrocyte tropomodulin (E-Tmod) capping its slow growing end (pointed end) [1–4].

As a TM-binding protein and the only capping protein at the pointed end in erythrocytes, E-Tmod plays a critical role in restricting the length of the short actin filament (F-actin) [1]. E-Tmod is a 41 kDa protein (E-Tmod41), which consists of N-terminal actin binding domain (E-Tmod_1–92_) and C-terminal actin binding domain with six leucine repeats [4]. E-Tmod41 binds to TM5/5b (35 nm) at 39–138 residues and the complex functions as a “molecular T ruler” metering off long actin filaments to short filaments of 37 nm [5]. Recently, a short E-Tmod isoform of 29 kDa (E-Tmod29) was discovered [6]. It lacks the N-terminal actin-binding domain but retains the C-terminal actin-binding domain. It can bind to TM5 or G-actin and is localized in the cytosol of erythrocytes. The expressions of E-Tmod41 and E-Tmod29 are driven by the alternative promoters of *E-Tmod* gene [7]. E-Tmod41 null mice display a mild sphero-elliptocytic anemia with osmotically fragile erythrocytes, due to the misregulation of F-actin lengths and a disrupted spectrin-actin lattice of membrane skeleton [8, 9]. In addition to E-Tmod, there are three members in Tmod family, neuronal Tmod (N-Tmod), ubiquitous Tmod (U-Tmod), and skeletal muscle Tmod (sk-Tmod) [10]. E-Tmod is the only Tmod isoform present in human and mouse mature erythrocytes [1]. But U-Tmod is found in erythroid progenitors and exists in the erythrocytes of TOT (Tropomodulin overexpressing transgenic) / E-Tmod^-/-^ mice. It may be due to the wide range of expression and the weak capping activity of U-Tmod [9].

The topology of membrane skeleton is formed during the development and maturation of erythrocytes. The synthesis and expression of major cytoskeletal proteins in erythrocyte membrane occur in an asynchronous manner and the remodeling of the membrane skeleton begins at a very early stage during erythrocyte development [11]. In the erythroid differentiation induced by erythropoietin, interleukin-3 (IL-3) and stem cell factor, etc., the gene expressions for many membrane skeleton proteins are significantly upregulated. In addition to chemical factors, the biomechanical force, fluid shear stress, has been found to contribute to the hematopoiesis in the embryos [12]. In adult, the reticulocytes have to circulate in the blood vessels before they become mature. Thus, these indicate that physical environment like fluid shear stress may also be involved in regulating of gene expressions of membrane skeleton proteins.

microRNAs (miRNAs) are the small non-coding RNA molecules containing about 22 nucleotides and play key roles in the regulation of gene expression. Many miRNAs, such as miR-451, miR-221/222, and let-7d, etc., are shown to be involved in the erythroid differentiation [13, 14]. There are mechano-sensitive miRNAs, such as miR-126, miR-23b, miR-10a, etc., which could be regulated by fluid shear stress in endothelial cells, vascular smooth muscle cells and macrophages [15, 16]. Therefore, it is possible that miRNAs may play roles in regulating the gene expressions of membrane skeleton proteins in the erythroid differentiation induced by fluid shear stress.

In the present study, we examined the effects of fluid shear stress on the erythroid differentiation, E-Tmod41 expression, and F-actin cytoskeleton remodeling. Our data showed that fluid shear stress could induce the erythroid differentiation and E-Tmod41 expression, thus contributing to the F-actin cytoskeleton remodeling. Furthermore, shear stress could upregulate E-Tmod41 expression by suppressing E-Tmod41-targeting miR-23b-3p and activating the alternative promoter upstream of exon 0.

## Materials and Methods

### Cell culture and animals

Mouse erythroleukemia (MEL) cell line derived from Friend virus-infected mice were maintained in RPMI 1640 medium containing 10% fetal bovine serum (FBS). Terminal erythroid differentiation was induced in MEL cells by adding 2% dimethyl sulphoxide (DMSO) in the culture medium for 24 hours. C57BL mice of 8 weeks old were paired. The vaginal plug was checked on the second day and counted as embryonic day 0.5 (E0.5). On E10.5, the pregnant mice were sacrificed by cervical dislocation. Embryonic erythroblasts were isolated from the yolk sacs embryos and maintained in IMDM medium containing 10% FBS, 10 units ml^-1^ penicillin, 10 μg ml^-1^ streptomycin, 0.2 mg ml^-1^ Fe-saturated transferrin, and 0.5 mg ml^-1^ ascorbic acid. The animal protocol was approved by the Ethical Committee of Peking University Health Science Center.

### Cell exposure to shear stress

MEL cells or erythroblasts were plated in a 6-cm Corning petri dish and the dish was placed in a cone-plate shearing system (Fig A in S1 File) [17, 18]. When the cone spins, the cells will be subjected to shear stress. The shear stress, τ, can be calculated based on the equation, *τ* = *μω*/ *α*, where *μ* is the viscosity of the cell suspension (2.21 mPa∙s), is the angular velocity, and *α* is the angle of the cone (0.5°). The shear stress was set to 5 dyn/cm^2^, which is the shear stress yielded in the dorsal aorta of E10.5 embryos [19].

### Flow cytometry

Cells were fixed in 4% paraformaldehyde, washed with phosphate buffered saline (PBS), and incubated with 0.1% Triton X-100 for 5 min. After blocked in 1% bovine serum albumin (BSA) for 30 min at room temperature, the cells were incubated with rhodamine phalloidin (Cytoskeleton Inc., USA) for 20 min in the dark at 37°C. Then the cells were resuspended in 300 μl PBS and analyzed in a BD FACS Calibur (USA). The mean fluorescent intensity was measured to represent the F-actin content in the cells.

### Laser scanning confocal microscopy

The cells were stained with rhodamine phalloidin following the procedures mentioned above. The nuclei were counterstained with 4',6-diamidino-2-phenylindole (DAPI, Beyotime Biotechnology, China) for 10 min and then washed with PBS. The samples were resuspended in the mixture of PBS and glycerol (v/v, 1:1) and placed into a coverglass bottom dish. The cells were observed with a laser scanning confocal microscope (Leica TCS SP8 MP FLIM, Germany). The mean fluorescent intensity of the images were calculated by using ImageJ 1.46r software (National Institutes of Health, USA).

### RNA isolation and Quantitative RT-PCR

Total RNA was extracted from cells using TRIzol reagent (Invitrogen, USA) according to the manufacturer’s instructions. Two microgram of total RNA was reverse transcribed to cDNA using a ReverAid First Strand cDNA Synthesis Kit (Thermo, USA). Real-time PCR was performed on a Mx3000 Multiplex Quantitative PCR system (Stratagene, USA) using Brilliant II SYBR Green QPCR Master Mix (Agilent Technologies, USA). The sequences of the primers used for E-Tmod41 were as follows: forward: 5’-GAC ACA GCC TCA CAC AAT GT-3’; reverse: 5’-CTT GGT GGT CTG ATC CTT CT-3’. The sequences of the primers for the markers of erythroid differentiation, β-major globin (*Hbb-b1*), glycophorin A (*GYPA*), and *GATA1* [20, 21], and miR-23b-3p host gene, *2010111I01Rik*, were listed in Table A in S1 File. GAPDH (forward: 5’-ACC ACA GTC CAT GCC ATC AC-3’; reverse: 5’- TCC CCA CCC TGT TGC TGT A-3’) was used as an internal control. A relative fold change in the gene expression was calculated using the method of 2^-△△CT^.

### Western blot analysis

Cells were lysed in RIPA buffer and centrifuged at 12000×*g* for 5 min at 4°C. The supernatant was collected and the protein concentration was quantified by BCA assay (Applygen Technologies Inc., Beijing, China). Proteins (20 μg) were separated by SDS-PAGE, and then transferred onto nitrocellulose membranes. The membranes were incubated with anti-E-Tmod41 antibody (prepared by AbMax Biotechnology Co., Ltd, Beijing, China), anti-glycophorin A antibody (Beijing Biosynthesis Biotechnology Co. Ltd, Beijing, China), anti-GAPDH antibody, or anti-β-tubulin (Santa cruz Biotech., USA), followed by HRP-conjugated goat anti-rabbit or mouse IgG. The signals were detected by using an Enhanced Chemiluminescence Detection (ECL) kit (Evergreen, Beijing, China).

### Adenovirus infection

MEL cells (1×10^6^ cells) were infected with adenovirus, Ad-E-Tmod41 and Ad-Null (SinoGenoMax, Beijing, China) at 0, 25, 50, 75, and 100 MOI for 48 hours. The cells were either lyzed for protein extraction or fixed in 4% paraformaldehyde for F-actin content analysis.

### siRNA, mimic, and inhibitor transfection

Small interfering RNA (siRNA) specific to E-Tmod41 was designed using Invitrogen Block-iT RNAi Designer. The sequence is 5’-GGA AUU UAA GGA CCG AGA A-3’. The mimic and inhibitor of miR-23b-3p were purchased from RiboBio (Guangzhou, China). MEL cells were transfected with siRNA, mimic, or inhibitor (50 and 100 nM) by using riboFECT CP transfection kit (RiboBio, Guangzhou, China). Transfected cells were harvested at 24 hours for mRNA extraction or at 48 hours for protein isolation.

### MicroRNA array analysis

MEL cells sheared for 12 hours and the unsheared controls (3 pairs) were harvested and lysed in TRIzol reagent (Life technologies, Carlsbad, CA). After RNA extraction and quality analysis, miRNAs were labeled and hybridized on the miRCURY LNA Array (v.18.0, Exiqon, Danmark) at KangChen Bio-tech Inc. (Shanghai, China). The images were analyzed and data were extracted using GenePix Pro 6.0 software (Axon). The microarray data was validated by using TaqMan Assay (Life technologies).

### Plasmid construction, site-directed mutagenesis, and dual luciferase assay

P_E0_ (~700 bp) and P_E1_ (~1000 bp) promoter sequences were amplified from mouse genomic DNA and inserted to pGL3 basic vector (Promega, USA) at the multiple cloning sites, respectively. The E-Tmod 3’-untranslated region (3’UTR) was amplified from mouse cDNA and subcloned to pGL3 control vector at XbaI site. The mutagenesis of potential miR-23b-3p targeting site was introduced by using site-directed mutagenesis kit (Tiangen Biotech., Beijing, China). For transfection, 1 μg of plasmid and 100 ng of renilla luciferase vector were co-transfected into MEL cells by FuGENE 6 (Promega, USA). Cells were collected after 48 hours for the detection of luciferase activity by the dual-luciferase reporter assay system (Promega, USA). For miRNA study, pGL3 control vector containing wild type or mutated E-Tmod 3’UTR were co-transfected into MEL cells with miR-23b-3p mimics (50 nM) or control mimics. Then the cells were collected after 24 hours for the dual luciferase assay.

### Statistical analysis

All experiments were performed in duplicate with data averaged from at least three independent experiments. The data are presented as the mean ± SEM. Direct comparisons were made using paired or unpaired Student’s *t*-test, and multiple group comparisons were made using one-way analyses of variance (ANOVA). Statistical significance was defined as *p*<0.05, 0.01, or 0.001 (indicated as *, **, or ***, respectively). Prism 5.0 software (GraphPad, Inc., USA) was used for data analyses.

## Results

### Fluid shear stress induces erythroid differentiation and F-actin cytoskeleton remodeling in MEL cells

**Fig 1:**
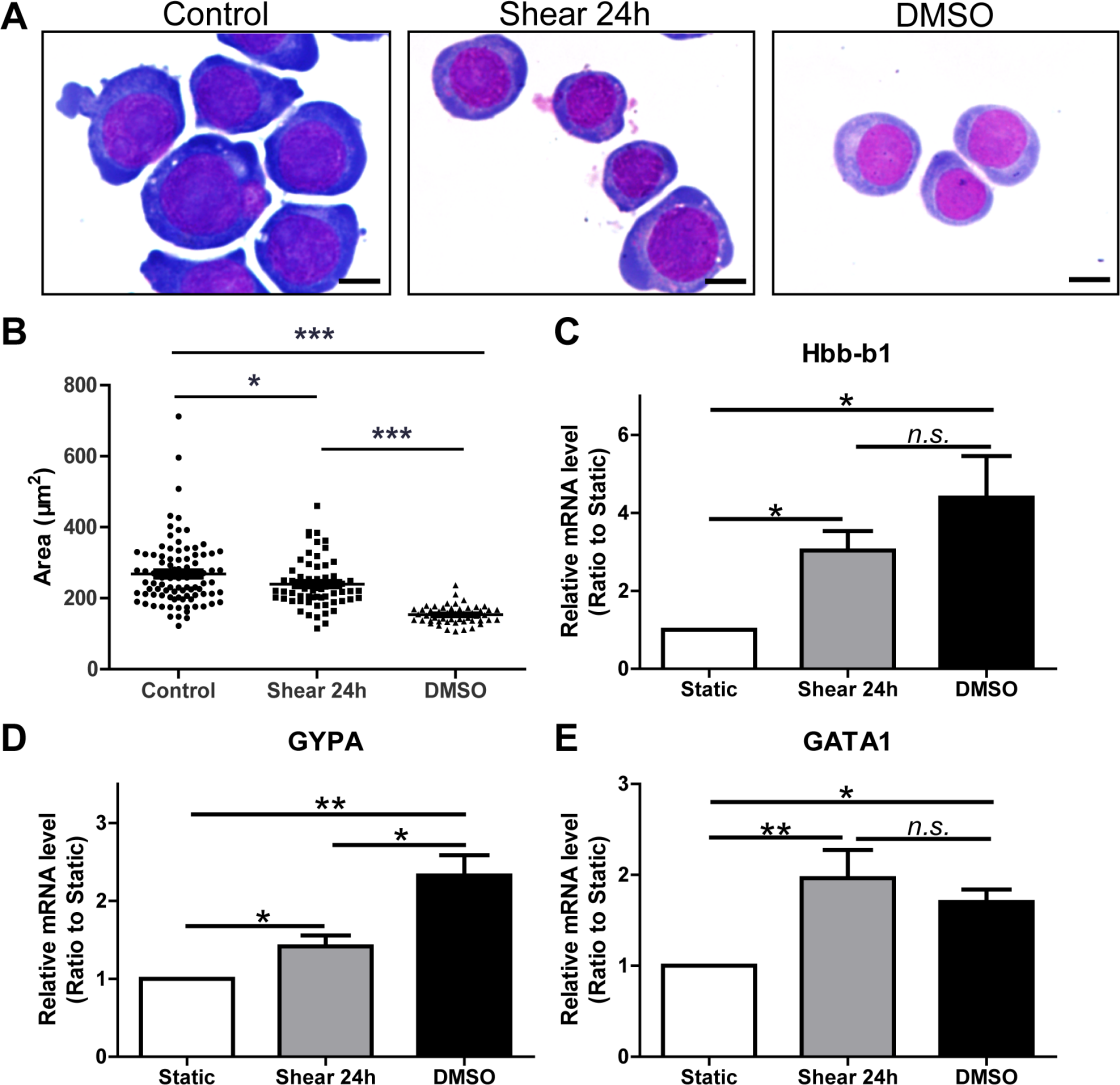
Fluid shear stress induced erythroid differentiation in MEL cells. (A) Representative images of Wright-Giemsa staining on MEL cells treated with shear stress (middle panel) and 2% DMSO (right panel) and control cells (left panel). Bars represent 10 μm. (B) The areas of the cells in different groups. (C-E) The mRNA expression levels of markers for erythroid differentiation, *Hbb-b1* (C), *GYPA* (D), and *GATA1* (E), as detected by quantitative RT-PCR. *: *p*<0.05, ***: *p*<0.001. *n*.*s*.: no significance.

We employed MEL cell, a cell model commonly used for the study of erythroid differentiation [22, 23], to examine the effects of fluid shear stress on the erythroid differentiation and cytoskeleton remodeling. A cone-plate shearing device was developed to generate the laminar shear stress of 5 dyn/cm^2^ (Fig A in S1 File), mimicking the blood flow in the aortas of embryos at embryonic day 10.5 [19]. MEL cells treated with 2% DMSO served as the positive control for erythroid differentiation. Wright-Giemsa staining (Fig 1A) showed that the dark blue color became reddish in the cytosol of MEL cells treated with shear stress or DMSO and that the nuclei became condensed. Further analysis showed that their cellular area significantly decreased as compared to control (Fig 1B). Quantitative RT-PCR data showed that markers for erythroid differentiation, *Hbb-b1*, *GYPA*, and *GATA1*, were all significantly upregulated by both shear stress and DMSO (Fig 1C–1E). These data indicate that, although it was less competent than DMSO, fluid shear stress indeed induced the erythroid differentiation in MEL cells.

**Fig 2:**
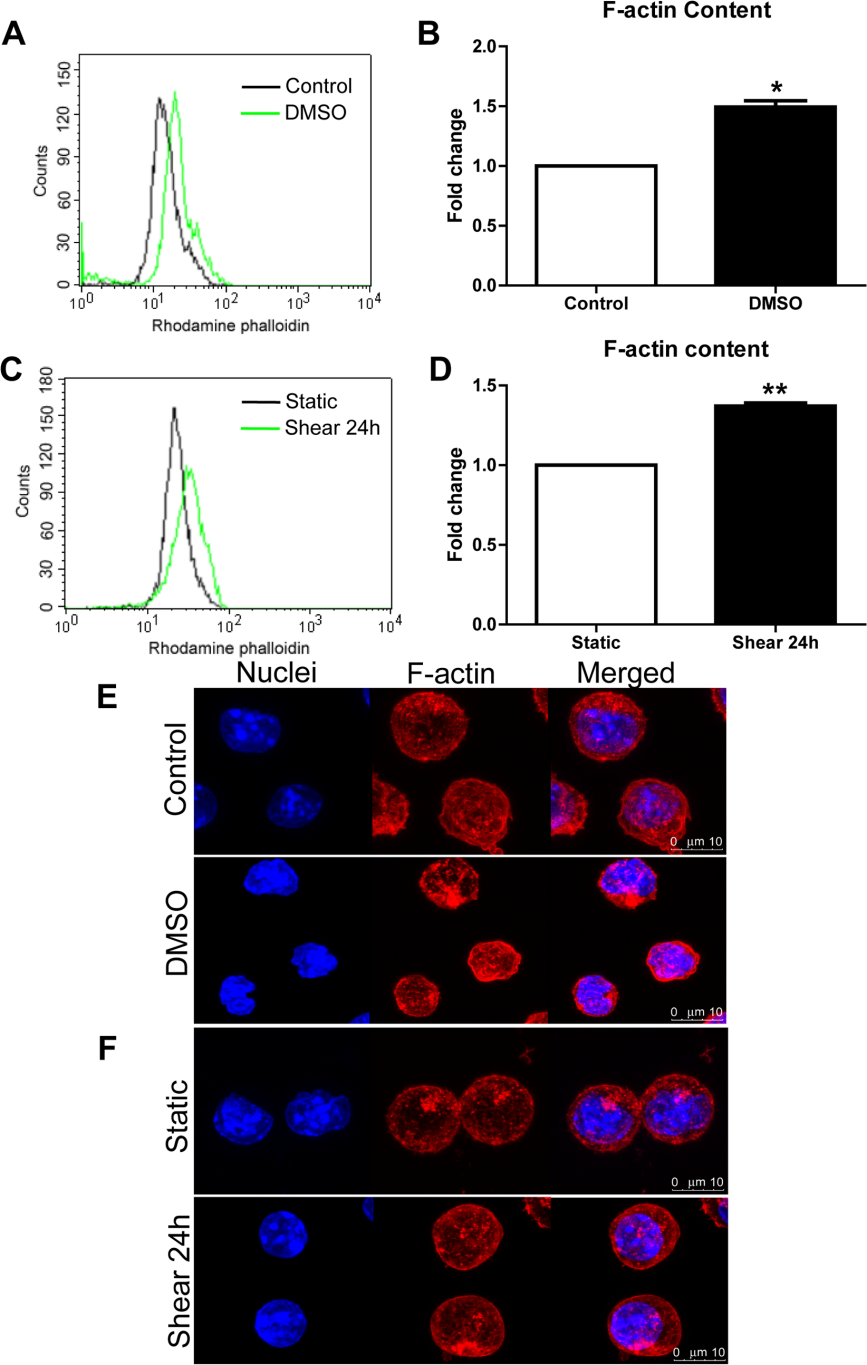
Fluid shear stress induced F-actin cytoskeleton remodeling in MEL cells. (A-D) Representative flow cytometry images for MEL cells treated with 2% DMSO (A) and shear stress (C) for 24 h and stained with rhodamine phalloidin. The fold changes in F-actin content were shown in (B) and (D), respectively. As compared to control or static, *: *p*<0.05, **: *p*<0.01. (E, F) The confocal images for cells treated with 2% DMSO (E) and shear stress (F).

Since the morphological change is accompanied with F-actin cytoskeleton remodeling during erythroid differentiation, we examined the F-actin content in MEL cells treated with DMSO and shear stress. Flow cytometry analysis showed that the F-actin content increased ~1.5 fold in both DMSO- and shear stress-treated MEL cells (Fig 2A–2D). Confocal images also showed the similar results (Fig 2E and 2F).

### Fluid shear stress upregulates E-Tmod41 in MEL cells

**Fig 3:**
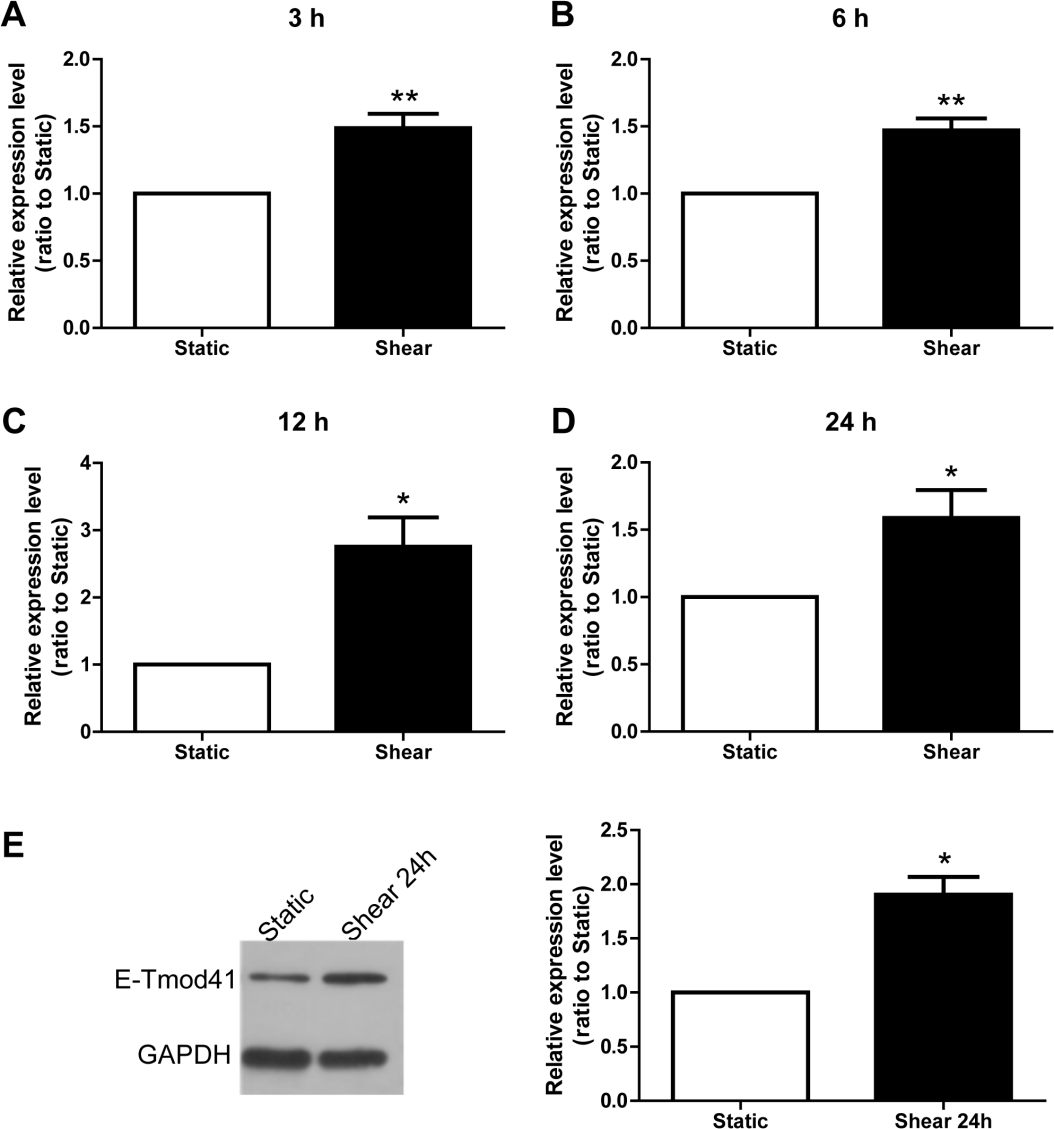
Fluid shear stress upregulated E-Tmod41 expression in MEL cells. (A-D) E-Tmod41 mRNA expression levels in MEL cells sheared for 3 h (A), 6 h (B), 12 h (C) and 24 h (D) as detected by quantitative RT-PCR. (E) The E-Tmod41 protein level in MEL cells sheared for 24 h as detected by Western blot (left panel) and its quantified data (right panel). GAPDH served as an internal control. As compared to static, *: *p*<0.05.

As the capping protein at the pointed end of F-actin, E-Tmod41 is critical for F-actin polymerization and stability. It may play a role in the F-actin cytoskeleton remodeling induced by fluid shear stress. Therefore, we examined E-Tmod41 expression in shear stress-treated MEL cells. Quantitative RT-PCR showed that E-Tmod41 mRNA expression was upregulated by 3 h of shearing (Fig 3A). As the shearing time extended, E-Tmod41 mRNA level increased and reached the highest level at 12 h (Fig 3B–3D). As detected by Western Blot, E-Tmod41 protein level did not change at 12 h (data not shown) but markedly increased 2 fold at 24 h (Fig 3E). The data indicate that fluid shear stress could upregulate E-Tmod41 expression.

### E-Tmod41 regulates F-actin content in MEL cells

**Fig 4:**
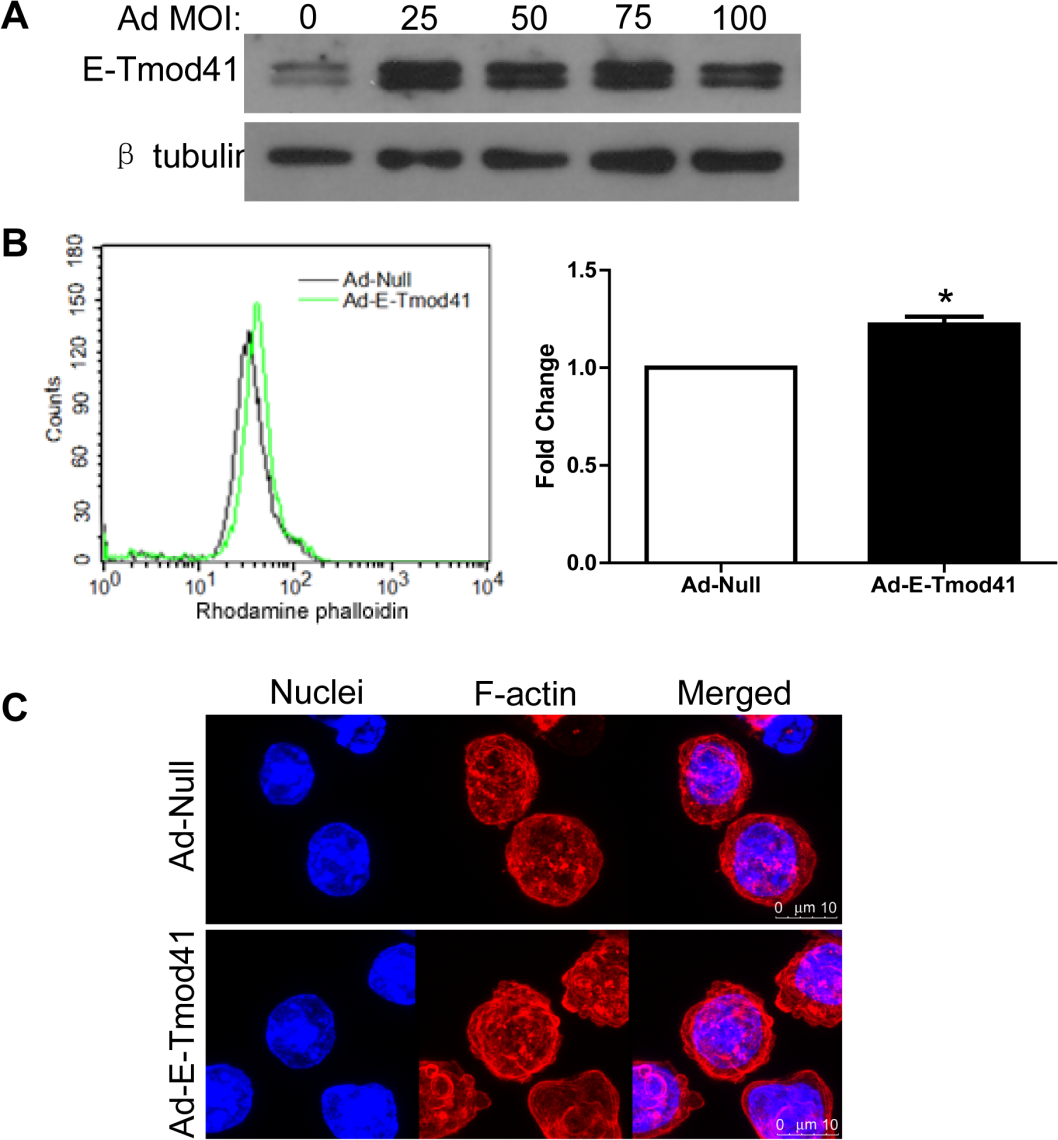
Overexpression of E-Tmod41 increased F-actin content in MEL cells. (A) E-Tmod41 expression in MEL cells infected with adenovirus, Ad-E-Tmod41, at 0, 25, 50, 75 and 100 MOI. β-tubulin served as an internal control. (B) Representative flow cytometry image (left panel) and the fold change of F-actin content (right panel) for MEL cells infected with Ad-E-Tmod41 and Ad-Null (50 MOI) and stained with rhodamine phalloidin. As compared to Ad-Null, *: *p*<0.05. (C) The F-actin images taken by confocal microscope (C).

**Fig 5:**
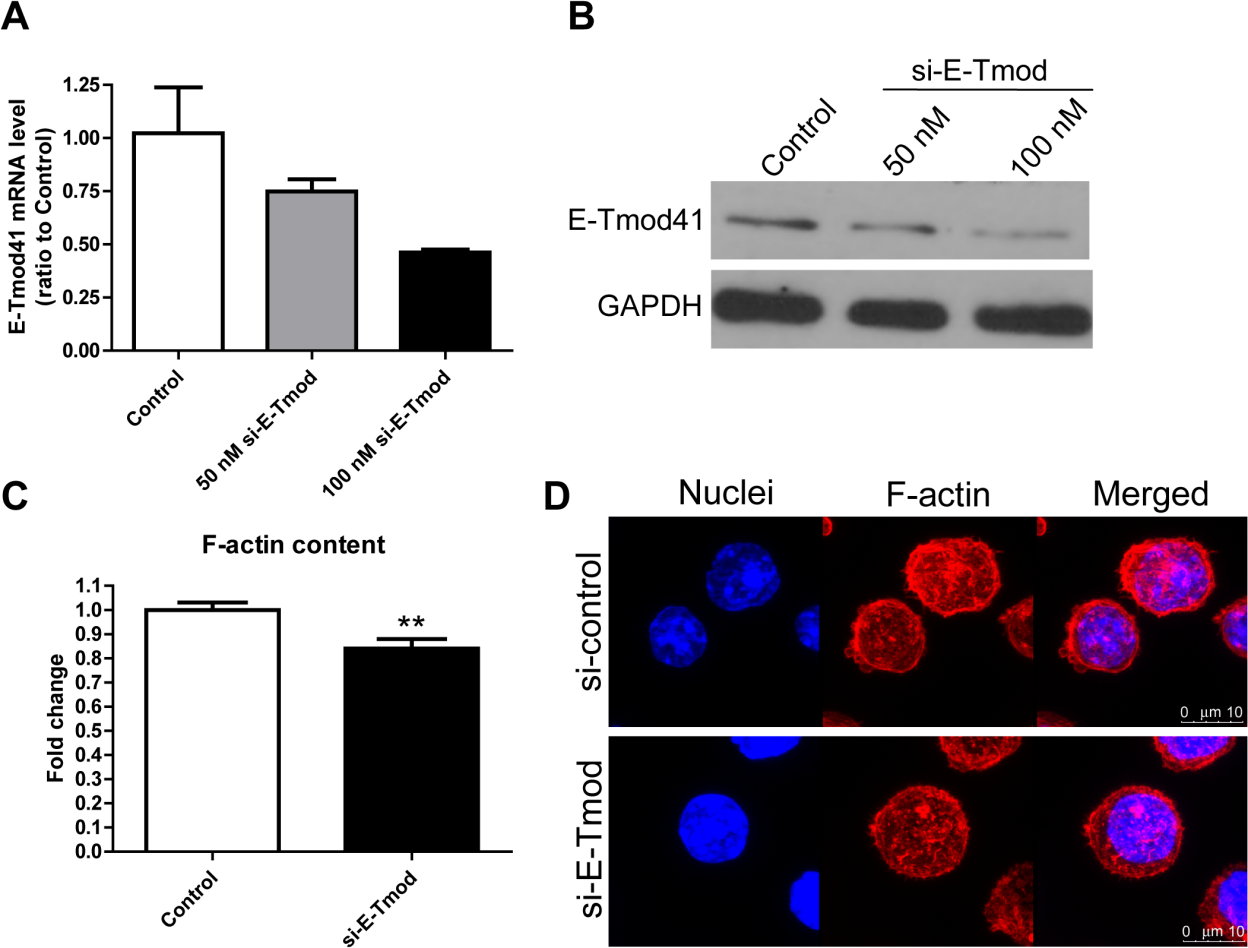
Knockdown of E-Tmod41 inhibited F-actin content in MEL cells. (A, B) E-Tmod41 mRNA level (A, N = 2) and protein level (B) in MEL cells transfected with si-E-Tmod. (C, D) The flow cytometry data (C) and the confocal images (D) for MEL cells transfected with 100 nM si-E-Tmod and control siRNA and stained with rhodamine phalloidin. As compared to control, **: *p*<0.01.

The upregulation of E-Tmod41 expression by shear stress may contribute to the F-actin cytoskeleton remodeling. We next performed the gain or loss of function experiments to study the effect of E-Tmod41 in F-actin remodeling. E-Tmod41 was over-expressed by adenovirus infection (Fig 4A). Both flow cytometry and confocal microscopy showed that the F-actin content was significantly increased in MEL cells infected by 50 MOI Ad-E-Tmod41 (Fig 4B and 4C). On the contrary, when E-Tmod41 was knocked down by E-Tmod41-specific siRNA (Fig 5A and 5B), the F-actin content was greatly reduced (Fig 5C and 5D). These findings demonstrate that E-Tmod41 affected the F-actin content in MEL cells, which may mediate the F-actin cytoskeleton remodeling induced by shear stress. To explore whether the expression of E-Tmod41 would affect the erythroid differentiation, we detected the expressions of *Hbb-b1*, *GYPA* and *GATA1* in MEL cells with E-Tmod41 overexpression or suppression. But quantitative RT-PCR data showed that none of these markers changed (Figs A and B in S1 File).

### Fluid shear stress suppresses the expression of an E-Tmod-targeting miRNA, miR-23b-3p

**Fig 6:**
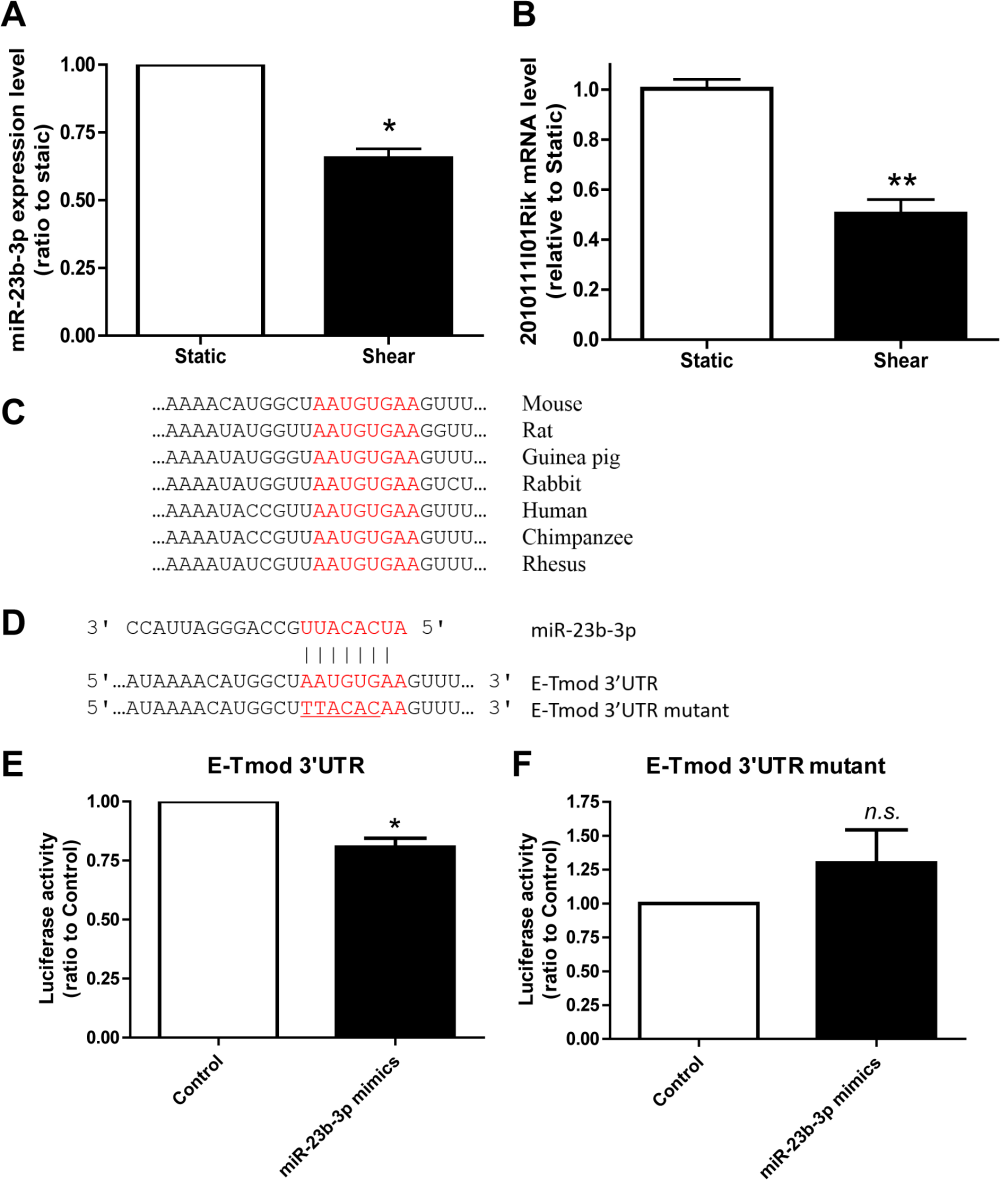
Fluid shear stress suppressed the expresson of miR-23b-3p and E-Tmod mRNA 3’UTR is the target of miR-23b-3p. (A, B) The expressions of miR-23b-3p and its host gene, *2010111I01Rik*, as detected by microRNA array analysis (A) and quantitative RT-PCR (B). (C) The putative binding site of miR-23b-3p (in red) is highly conservative in mammals. (D) Wild type E-Tmod 3’UTR with miR-23b-3p binding site and its mutant form (bottom). (E, F) The luciferase activities in MEL cells cotransfected with miR-23b-3p mimic and the luciferase reporter plasmids carrying either wild type (E) or mutant form of E-Tmod 3’UTR (F). As compared to control, *: *p*<0.05, *n*.*s*.: no significance.

miRNAs play important roles in hematopoiesis and there are many miRNAs that could be regulated by fluid shear stress. So we collected MEL cells sheared for 12 h and performed miRNA microarray analysis. Data showed that 24 miRNAs were upregulated and 58 miRNAs were down-regulated >1.5 fold (with *p* value <0.05) by shear stress (Table B in S1 File). Among the down-regulated miRNAs, miR-23b-3p was predicted to target E-Tmod mRNA 3'UTR by TargetScan and Pictar. Quantitative RT-PCR data showed that, in addition to miR-23b-3p, fluid shear stress could also suppress the expression of miR-23b-3p’s host gene, *2010111I01Rik* (Fig 6A and 6B). Importantly, *2010111I01Rik* is not a target of miR-23b-3p (Fig D in S1 File).

Sequence analysis showed that the targeting site of miR-23b-3p on E-Tmod 3’UTR is highly conserved in mammals (Fig 6C). To test whether miR-23b-3p targets E-Tmod, two reporter vectors were constructed with a luciferase-coding sequence followed by wild type or mutant E-Tmod 3’UTR (Fig 6D), which were named as pGL3-E-Tmod 3’UTR and pGL3-E-Tmod 3’UTR mutant, respectively. Dual luciferase assay demonstrated that the luciferase activity of pGL3-E-Tmod 3’UTR was reduced by miR-23b-3p mimic, while that of pGL3-E-Tmod 3’UTR mutant did not change (Fig 6E and 6F). The data suggest that fluid shear stress downregulated the expression of miR-23b-3p and that miR-23b-3p was an E-Tmod targeting miRNA.

### miR-23b-3p regulates E-Tmod41 expression and F-actin content in MEL cells

**Fig 7:**
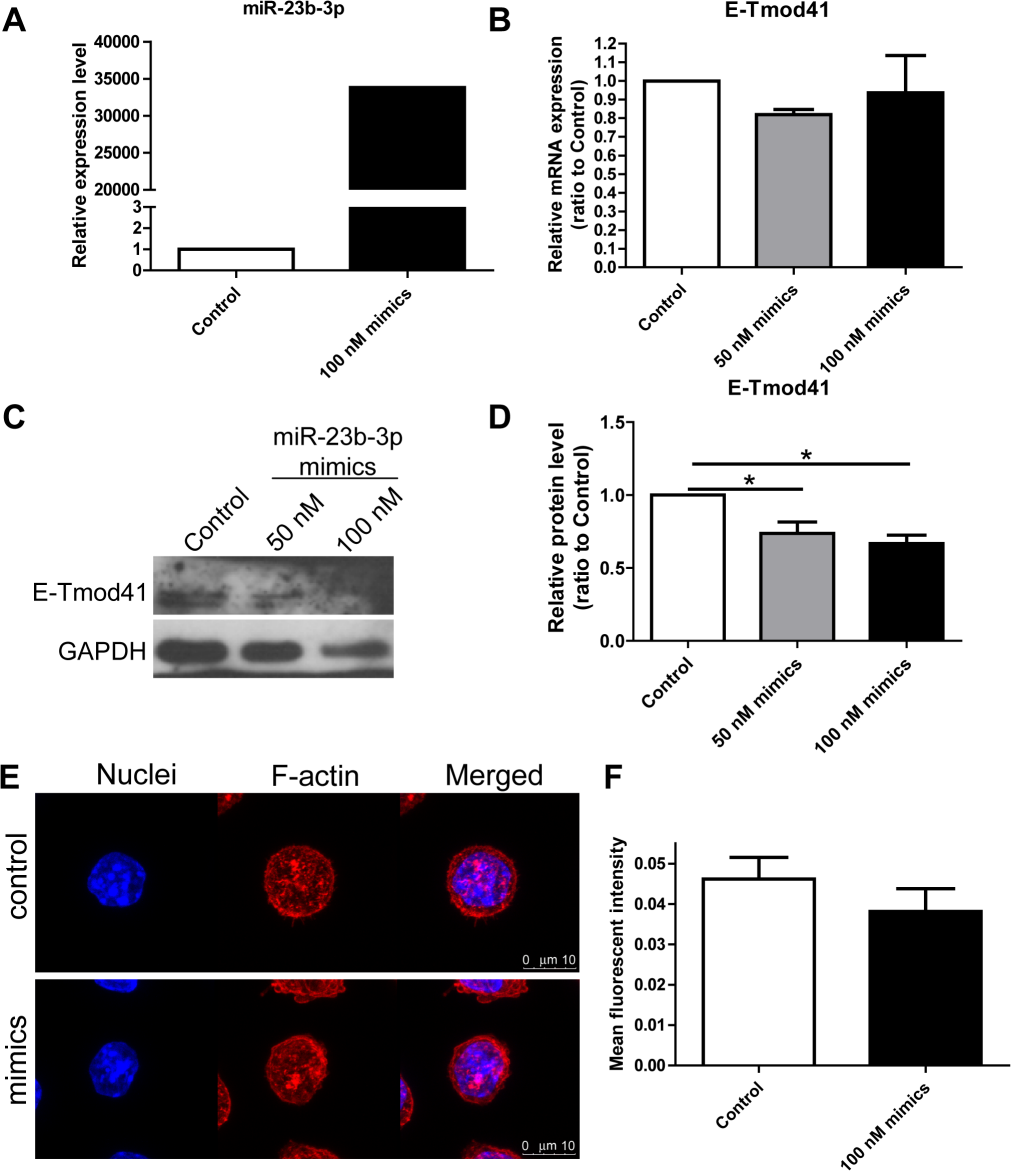
Overexpression of miR-23b-3p downregulated E-Tmod41 expression and contributed to F-actin cytoskeleton remodeling. (A) The expression of miR-23b-3p in MEL cells transfected with miR-23b-3p mimic (100 nM) was verified by quantitative RT-PCR. (B, C) E-Tmod41 mRNA levels (B) and protein levels (C) in MEL cells transfected with miR-23b-3p mimic (50 and 100 nM). GAPDH served as an internal control. The quantified data was shown in (D). As compared to control, *: *p*<0.05. (E) The confocal images for MEL cells transfected with miR-23b-3p mimic and control mimic and stained with rhodamine phalloidin. The mean fluorescent intensities of the images were quantified and shown in (F).

**Fig 8:**
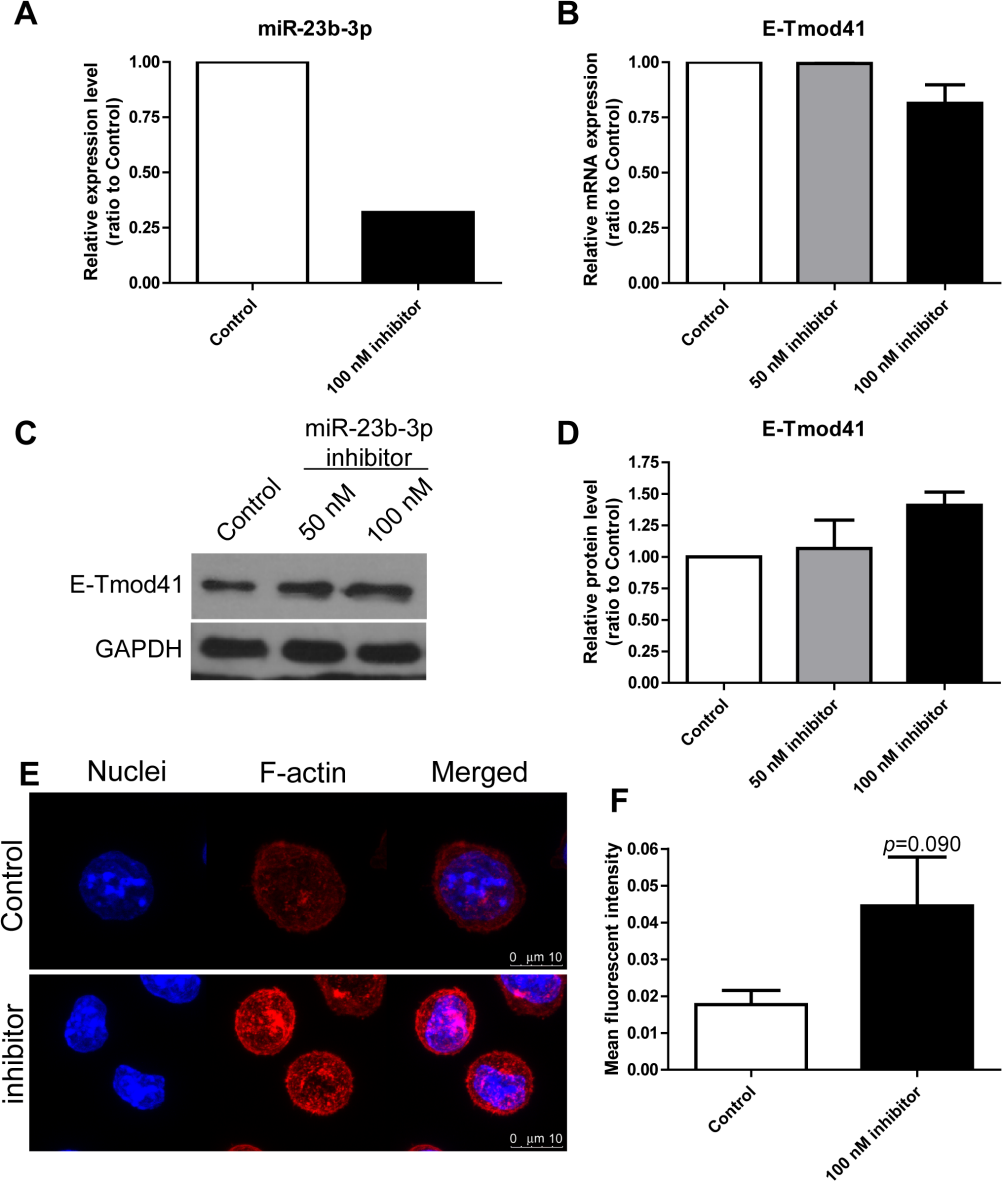
Knockdown of miR-23b-3p upregulated E-Tmod41 expression and contributed to F-actin cytoskeleton remodeling. (A) The expression of miR-23b-3p in MEL cells transfected with miR-23b-3p inhibitor (100 nM) was verified by quantitative RT-PCR. (B, C) E-Tmod41 mRNA levels (B) and protein levels (C) in MEL cells transfected with miR-23b-3p inhibitor (50 and 100 nM). GAPDH served as an internal control. The quantified data was shown in (D, N = 2). (E) The confocal images for MEL cells transfected with miR-23b-3p inhibitor and control inhibitor and stained with rhodamine phalloidin. The mean fluorescent intensities of the images were quantified and shown in (F).

Since miR-23b-3p was shown to be an E-Tmod targeting miRNA, we next examined whether miR-23b-3p could regulate E-Tmod41 expression and thus affect F-actin content in MEL cells. miR-23b-3p was overexpressed or knocked down with mimic or inhibitor (Figs 7A and 8A). Quantitative RT-PCR showed that mRNA level of E-Tmod41 was not changed by miR-23b-3p mimic or inhibitor (Figs 7B and 8B). But the E-Tmod41 protein level was decreased or increased by mimic (Fig 7C and 7D) or inhibitor (Fig 8C and 8D). F-actin was stained in MEL cells transfected with miR-23b-3p mimic or inhibitor. Confocal microscope images were taken (Figs 7E and 8E) and the mean fluorescent intensities (representing F-actin content) were measured. Data showed that miR-23b-3p mimic reduced F-actin content in MEL cells (Fig 7F), while miR-23b-3p inhibitor had the opposite effect (Fig 8F).

### Fluid shear stress regulates *E-Tmod* alternative promoters

**Fig 9:**
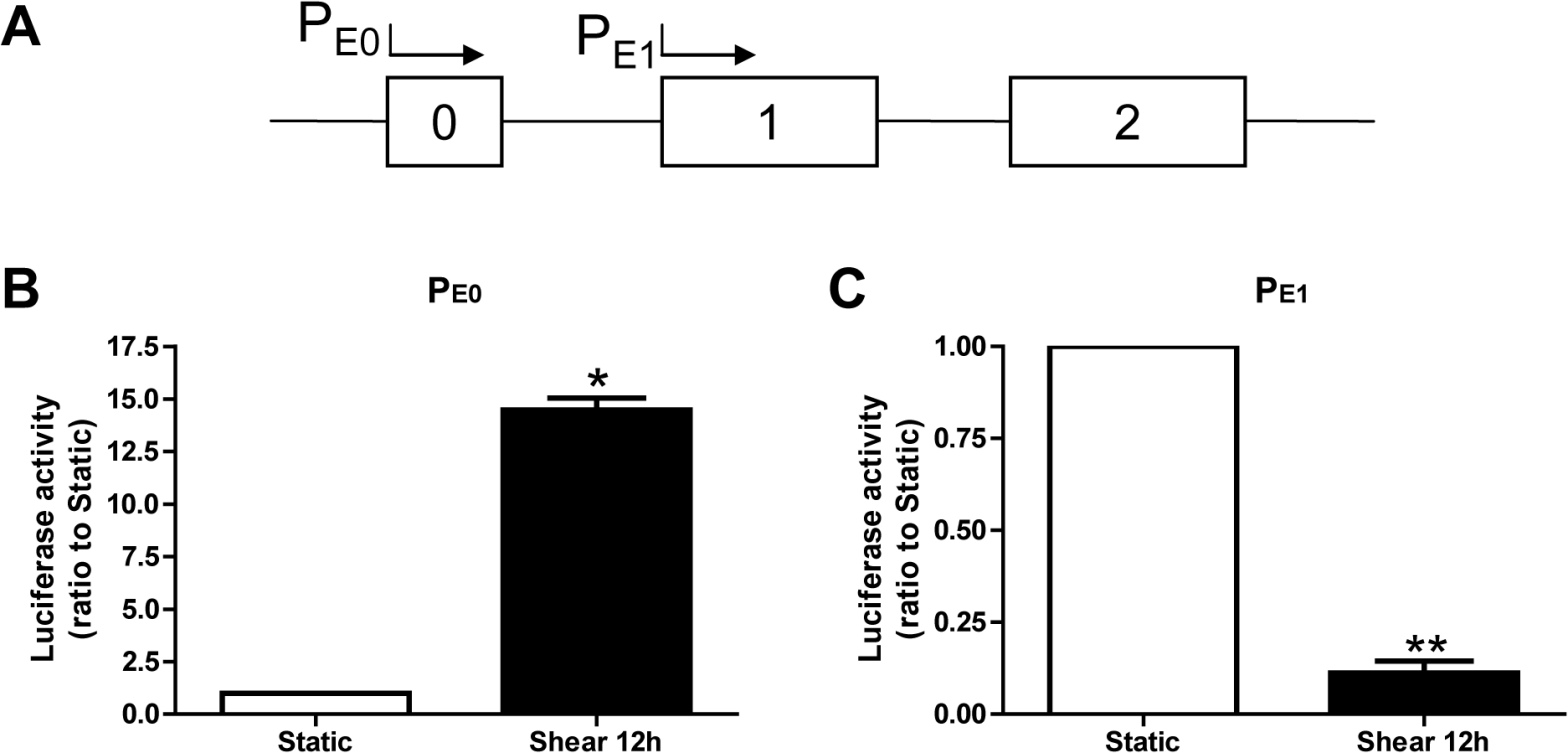
Fluid shear stress regulated the activities of alternative promoters of *E-Tmod*. (A) Schematic diagram of alternative promoters of *E-Tmod*. Boxes represent exons and lines represent introns. (B, C) The luciferase activities in MEL cells transfected with luciferase reporter plasmids driven by P_E0_ (B) or P_E1_ (C) promoters and subjected to shear stress for 12 h. As compared to static, *: *p*<0.05, **: *p*<0.01.

The expression of *E-Tmod* gene was driven by two alternative promoters [6, 7]. One is located up-stream of non-coding exon 0 (E0), named as promoter E0 (P_E0_), the other is located up-stream of exon 1 (E1), named as promoter E1 (P_E1_) (Fig 9A). Our previous study showed that P_E0_ preferably regulated the transcription of E-Tmod41, while P_E1_ preferably regulated the transcription of E-Tmod29 [6]. Therefore, we constructed two reporter vectors, with the luciferase coding sequence driven by P_E0_ or P_E1._ MEL cells were transfected with two vectors, respectively, and then subjected to shear stress for 12 hours. Dual luciferase assay showed that P_E0_ activity was augmented over 10 fold by shear stress (Fig 9B), while P_E1_ activity was reduced to 1/10 of the control level (Fig 9C).

### Fluid shear stress induces erythroid differentiation and E-Tmod41 expression in mouse embryonic erythroblasts

**Fig 10:**
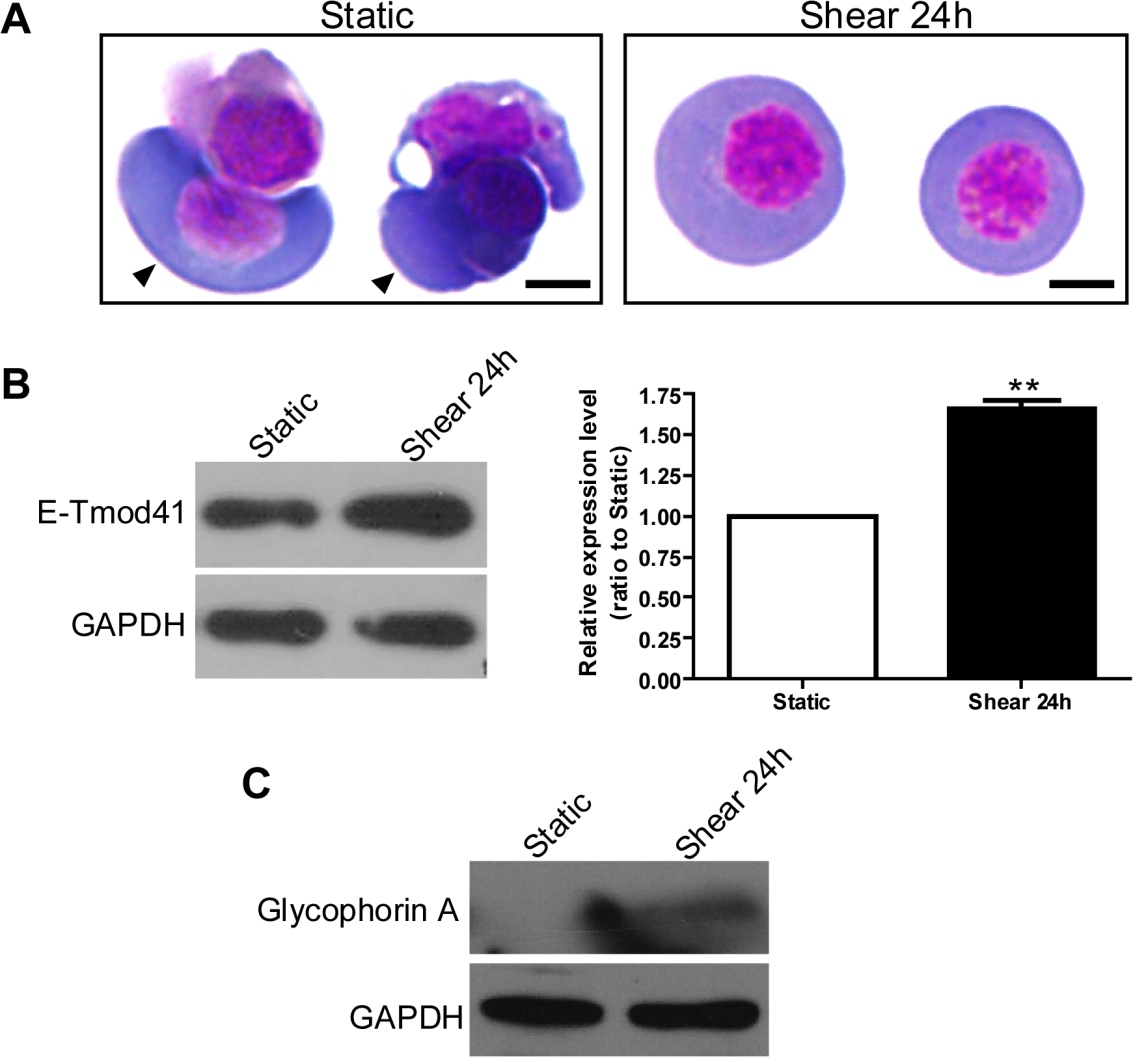
Fluid shear stress induced erythroid differentiation and upregulated E-Tmod41 protein expression in embryonic erythrocytes. (A) Representative Wright-Giemsa staining images for erythroblasts isolated from yolk sacs of day 10.5 embryos and subjected to shear stress for 24 h. The arrows in left panel indicate the erythroblasts. Bars represent 5 m. (B) The E-Tmod41 protein level was detected by Western blot (left panel) and the quantified result was shown in the right panel. As compared to static, **: *p*<0.01. (C) The protein level of glycophorin A were also detected by Western blot.

To test the results obtained in MEL cells, mouse erythroblasts were isolated from mouse E10.5 embryos and subjected to shear stress for 24 hours. Wright-Giemsa staining showed that un-sheared erythroblasts had the characteristics of polychromatic erythroblasts. After shearing, the dark blue in the cytosol became much lighter and the nuclei became condensed (Fig 10A). Western blot results demonstrated that the protein expressions of E-Tmod41 and glycophorin A were greatly upregulated in sheared erythroblasts (Fig 10B and 10C). These data suggests that shear stress could induce erythroid differentiation and E-Tmod41 expression in primary erythroblasts, which is consistent with the data obtained in MEL cells.

## Discussion

Previous efforts have proved that hemogenic endothelial cells could be induced to differentiate into hematopoietic cells both *in vivo* and *in vitro* by fluid shear stress in zebra fish and mouse [17, 24]. Furthermore, hematopoietic stem cells (HSCs) or multipotent progenitor cells circulate and differentiate in the embryonic blood vessels. Therefore, erythroid differentiation should also be regulated by shear stress. Here, by using both MEL cells and embryonic erythroblasts, we showed that fluid shear stress yielded in aortas of E10.5 embryos, could indeed induce the erythroid differentiation (Figs 1 and 10), which is consistent with Adamo’s observation [17]. The effect of fluid shear stress on erythroid differentiation may come from its regulation on the transcriptional factor Krüppel-like factor 2 (Klf2), which has been proved to play a role in erythropoiesis [25]. Our preliminary result showed that Klf2 mRNA level was significantly upregulated in MEL cells sheared for 6 hours (data not shown), which is consistent with previous studies.

Findings of flow cytometry and confocal microscopy analyses showed that MEL cells treated with both fluid shear stress and DMSO had increased F-actin cytoskeleton content as compared to the control cells (Fig 2). This means fluid shear stress contributes to F-actin cytoskeleton remodeling during erythroid differentiation. Our previous work showed that DMSO treatment could upregulate the expressions of E-Tmod41 and its transcription factor GATA1 in MEL cells. In this study, we found fluid shear stress could upregulate the expression of E-Tmod41 (Fig 3). Further studies with overexpression or knockdown of E-Tmod41 indicate that E-Tmod41 affects F-actin content (Figs 4 and 5), which may explain the mechanism for the effect of fluid shear stress on F-actin remodeling. E-Tmod29, an isoform of E-Tmod that can bind to TM and G-actin in the cytosol, was found downregulated in MEL cells after shearing (data not shown), suggesting that it may play a role in F-actin cytoskeleton remodeling. The downregulation of E-Tmod29 may free more TMs and G-actins, which are available for F-actin assembly.

As the capping protein at the pointed end of F-actin, E-Tmod and its family members play important roles in regulating F-actin structures. Recent studies are mainly focused on E-Tmod and U-Tmod due to their wide expressions. The abilities of U-Tmod that capping the pointed end of F-actin and binding to TMs (including α/βTM, TM5b, TM5NM1) are weaker than E-Tmod [10]. Therefore, U-Tmod is involved in the dynamic change of F-actin structure, and regulates the cell motility in endothelia [26] and insulin-stimulated GLUT4 exocytosis in adipocytes [27]; while E-Tmod is widely expressed in the terminally differentiated cells, such as erythrocytes, cardiomyocytes, and skeletal muscle cells, etc [28, 29]. U-Tmod was found in the hematopoietic stem and progenitor cells and plays an important role in terminal differentiation of fetal liver erythroid cells [9, 30]. During the erythroid differentiation, U-Tmod was downregulated and E-Tmod41 was upregulated, finally, leaving E-Tmod41 to be the sole protein that caps the pointed end of F-actin [9, 30]. This information suggests that U-Tmod may be downregulated during the erythroid differentiation induced by fluid shear stress, which would provide more pointed ends for E-Tmod41 to cap and accelerate F-actin remodeling. In addition, our data suggested that E-Tmod41 does not affect the erythroid differentiation (Figs A and B in S1 File). This is consistent with previous findings that E-Tmod null mice only have a mild spherocytic elliptocytosis and seem to have no defect in erythroid differentiation [9].

miRNAs are involved in both erythroid differentiation [14] and the mechanical responses in endothelial cells, vascular smooth muscle cells, and macrophages [15, 16]. By using microRNA array, we found that many miRNAs are differentially regulated by fluid shear stress in MEL cells. Among them, mechanosensitive miR-23b-3p, which was proved to be an E-Tmod targeting miRNA, was found suppressed by shear stress (Fig 6). The suppression of miR-23b-3p resulted in the upregulation of E-Tmod41 protein expression and contributed to F-actin remodeling in MEL cells (Figs 7 and 8). We noticed that miR-23b was found upregulated by pulsatile shear stress in human umbilical endothelial cells and participated in the regulation of cell proliferation [31]. The contradictory results may come from the different responses of one miRNA to different flow patterns in various cell types. It was reported that miR-23b could enhance the connections between breast cancer cells and that the inhibition of miR-23b enhanced their migration and deformation abilities [32]. MiR-23b was found to directly target p21-activated kinase 2 (PAK2) and increase the phosphorylation of myosin II. In addition, miR-23b was found to target many genes that participate in cell cytoskeleton remodeling [33]. These studies suggest that miR-23b can regulate cytoskeleton and deformation of cells. In our case, it contributes to F-actin cytoskeleton remodeling in sheared MEL cells by targeting E-Tmod41.

Our data showed that fluid shear stress could activate P_E0_ promoter to promote E-Tmod41 expression and suppress P_E1_ promoter to inhibit E-Tmod29 expression (Fig 9). This is consistent with our previous observations that P_E0_ activity is increased and becomes dominant in maturating reticulocytes, while P_E1_ activity is high in undifferentiated erythroblasts [6]. The differential regulations of E-Tmod41 and E-Tmod29 by fluid shear stress would be beneficial to F-actin cytoskeleton remodeling and morphological change in erythroid cells. Besides, it should be pointed out that, in our study of miRNA, the protein level of E-Tmod29 was not changed by miR-23b-3p (data not shown), which means E-Tmod41 but not E-Tmod29 is the target of miR-23b-3p. Therefore, the down-regulation of E-Tmod29 is mainly through the regulation of alternative promoters by fluid shear stress but not through miR-23b-3p.

In our study, we used the cone-plate shearing device to shear the cells. Since MEL cells and embryonic erythroblasts are suspending cells, the shear stress acting on them in the shearing device may be much more complicated as compared to adherent cells, e.g., hemogenic endothelial cells. In addition, we used the shear stress yielded in the aortas of E10.5 embryos, 5 dyn/cm^2^. But for the erythroblasts in bone marrows and reticulocytes circulating in the blood, the mechanical forces they sense (including shear stress and hydrostatic pressure) and their physical environments may not be the same as in the embryonic aortas. Therefore, it would be useful if we design devices mimicking the environments of bone marrow or blood vessels by microfluidics and micro-nano techniques.

In conclusion, by using our experiment setup, our findings suggest that fluid shear stress induced the differentiation and F-actin cytoskeleton remodeling in nucleated erythroblasts. Fluid shear stress upregulated E-Tmod41 expression in MEL cells and embryonic erythroblasts and the change of E-Tmod41 contributed to F-actin cytoskeleton remodeling. The upregulation of E-Tmod41 by shear stress could be mediated by two mechanisms: the inhibition of its targeting miRNA, miR-23b-3p, and the activation of its promoter, P_E0_. Our work would help us to better understand the factors that regulates erythroid differentiation, the molecular mechanisms for cytoskeleton remodeling in erythroid cells, and the mechanisms for the mechanical regulation of E-Tmod.

## Supporting Information

S1 File Fig A. The picture of cone-plate shearing device used in the study. The stepping motor, cone, and plate are labeled. Fig B. The mRNA expressions of markers for erythroid differentiation in MEL cells infected with Ad-E-Tmod41. (a) Hbb-b1; (b) GYPA; (c) GATA1. Fig C. The mRNA expressions of markers for erythroid differentiation in MEL cells transfected with E-Tmod41 specific siRNA. (a) Hbb-b1; (b) GYPA; (c) GATA1. Fig D. miR-23b-3p has no effect on the expression of its host gene, *2010111I01Rik*. Table A in S1 File. The primer sequences used for quantitative RT-PCR. Table B in S1 File. Shear vs Static 1.5 fold up and down regulated miRNAs. (DOC)
