# Supplementary material for: Fluid Shear Stress Upregulates E-Tmod41 via miR-23b-3p and Contributes to F-Actin Cytoskeleton Remodeling during Erythropoiesis
Source: PLoS One. 2015 Aug 26;10(8):e0136607. doi: 10.1371/journal.pone.0136607 (PMC4550387; doi:10.1371/journal.pone.0136607)
Supplement: S1 File — (DOC) [file pone.0136607.s001.doc]

**Supplementary data**


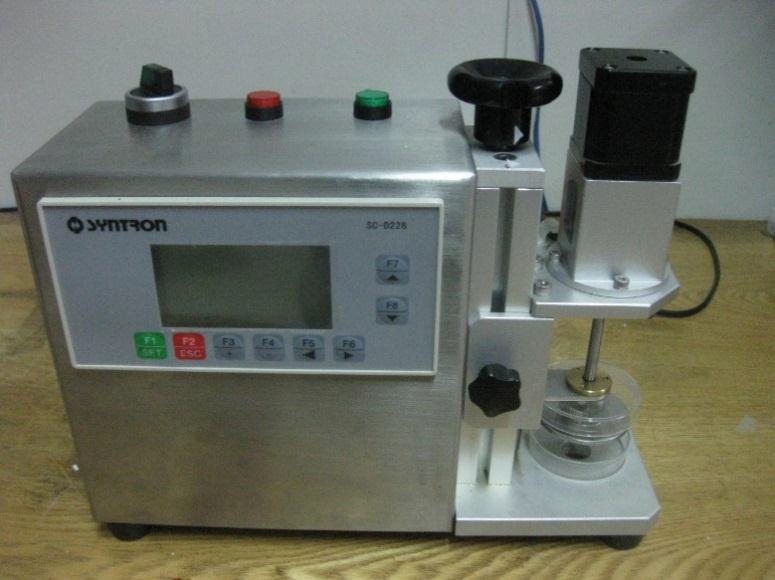


Stepping motor

Cone

Plate

**Fig A.** The picture of cone-plate shearing device used in the study. The stepping motor, cone, and plate are labeled.

**a**

**b**


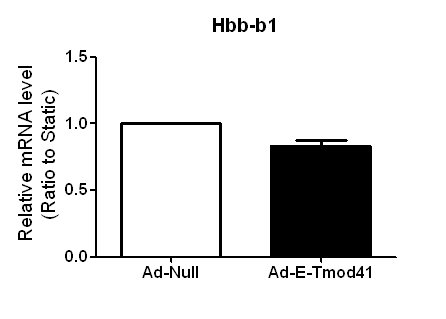

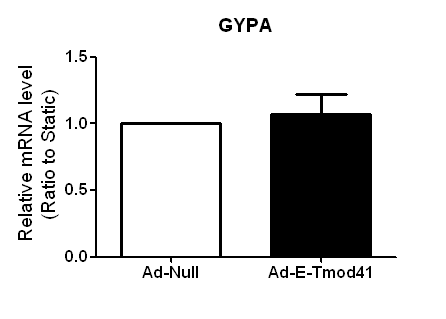


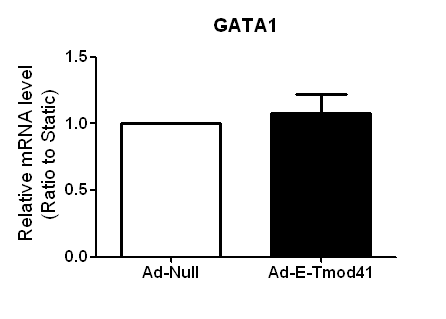


**c**

**Fig B.** The mRNA expressions of markers for erythroid differentiation in MEL cells infected with Ad-E-Tmod41. (a) Hbb-b1; (b) GYPA; (c) GATA1.


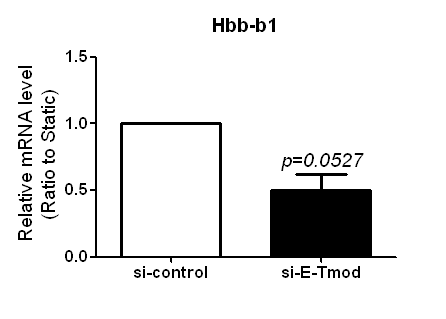

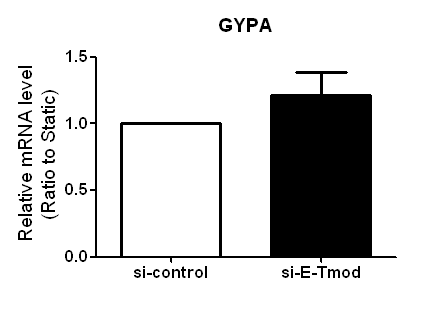


**a**

**b**


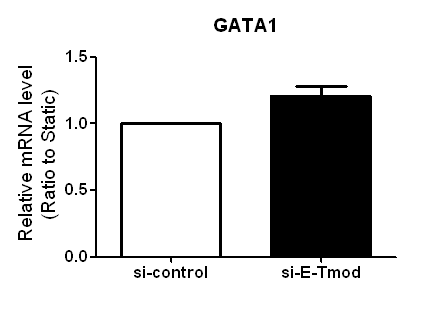


**c**

**Fig C.** The mRNA expressions of markers for erythroid differentiation in MEL cells transfected with E-Tmod41 specific siRNA. (a) Hbb-b1; (b) GYPA; (c) GATA1.

**Fig. D** miR-23b-3p has no effect on the expression of its host gene, *2010111I01Rik*.

**Table A.** The primer sequences used for quantitative RT-PCR .

| **Gene name** | **Primer sequence** |
| --- | --- |
| Hbb-b1 | Forward: 5’-AACGATGGCCTGAATCACTT-3’  Reverse: 5’-ACGATCATATTGCCCAGGAG-3’ |
| GYPA | Forward: 5’-ACTCCTGTGGTGGCTTCAAC-3’  Reverse: 5’-TCCTCCAATGTGTGGTGAGA-3’ |
| GATA1 | Forward: 5’-CCACTAAGGTGGCTGAATCC-3’  Revserse: 5’-AGGGCAGAATCCACAAACTG-3’ |
| 2010111I01Rik | Forward: 5’-GGACAACAGCCAGAATGGC-3’  Reverse: 5’-GATGCAGCTGCTCGAACTGT-3’ |

**Table B.** Shear vs Static 1.5 fold up and down regulated miRNAs.

| **Name** | **Fold change** |
| --- | --- |
| mmu-miR-673-3p | 9.646781727 |
| mmu-miR-3100-3p | 8.278076227 |
| mmu-miR-1971 | 7.996525802 |
| mmu-miR-3472 | 7.874717368 |
| mmu-miR-21a-3p | 7.8182857 |
| mmu-miR-185-3p | 6.232259834 |
| mmu-miR-874-3p | 5.377554462 |
| mmu-let-7e-3p | 4.185769249 |
| mmu-miR-291a-5p | 4.125401546 |
| mmu-miR-150-5p | 2.979302669 |
| mmu-miR-877-5p | 2.905808156 |
| mmu-miR-5616-3p | 2.771027171 |
| mghv-miR-M1-3-3p | 2.685116676 |
| mmu-miR-490-5p | 2.678804682 |
| mmu-miR-341-3p | 2.664165086 |
| mmu-miR-1931 | 2.642188867 |
| mmu-miR-3473b | 2.267326853 |
| mmu-miR-675-5p | 2.220335327 |
| mmu-miR-5120 | 2.036800103 |
| mmu-miR-5116 | 2.026454276 |
| mmu-miR-714 | 1.88213511 |
| mmu-miR-5128 | 1.748071568 |
| mmu-miR-302a-3p | 1.747699974 |
| mmu-miR-192-5p | 1.669633975 |
|  |  |
| mmu-miR-340-3p | 0.659375415 |
| mmu-miR-23b-3p | 0.611342337 |
| mmu-miR-148a-3p | 0.609222285 |
| mmu-miR-500-3p | 0.605824203 |
| mmu-miR-30d-3p | 0.599393532 |
| mmu-let-7f-1-3p | 0.588763247 |
| mmu-miR-3065-5p | 0.553895634 |
| mmu-miR-140-3p | 0.539521489 |
| mmu-miR-340-5p | 0.529604947 |
| mmu-miR-365-3p | 0.523987819 |
| mmu-miR-96-5p | 0.521618813 |
| mmu-miR-378a-5p | 0.520341248 |
| mmu-miR-142-5p | 0.517918003 |
| mmu-miR-26b-5p | 0.517615745 |
| mmu-miR-16-5p | 0.511787755 |
| mmu-miR-669e-5p | 0.507065774 |
| mmu-miR-212-3p | 0.502129003 |
| mmu-miR-144-3p | 0.500392727 |
| mmu-miR-106b-5p | 0.496852037 |
| mmu-miR-15a-5p | 0.494138127 |
| mmu-miR-547-3p | 0.488515416 |
| mmu-miR-146b-5p | 0.481339879 |
| mmu-miR-130b-3p | 0.47506261 |
| mmu-miR-29a-3p | 0.467007645 |
| mghv-miR-M1-8-5p | 0.464932654 |
| mmu-miR-196a-5p | 0.458220152 |
| mmu-miR-30e-3p | 0.453606111 |
| mmu-miR-25-3p | 0.452779879 |
| mmu-miR-191-5p | 0.448896998 |
| mmu-miR-1949 | 0.442101203 |
| mmu-miR-22-3p | 0.441838895 |
| mmu-miR-1960 | 0.435196769 |
| mmu-miR-101b-3p | 0.433546862 |
| mmu-miR-652-3p | 0.403622184 |
| mmu-miR-106b-3p | 0.400841423 |
| mmu-miR-20a-5p | 0.398455586 |
| mmu-miR-29a-5p | 0.38516404 |
| mmu-miR-302b-3p | 0.380824691 |
| mmu-miR-98-5p | 0.377429334 |
| mmu-miR-17-3p | 0.37672426 |
| mmu-miR-187-3p | 0.373554656 |
| mmu-miR-200b-3p | 0.368830101 |
| mmu-miR-98-3p | 0.367215661 |
| mmu-miR-186-5p | 0.362182436 |
| mmu-miR-101a-3p | 0.348560167 |
| mmu-miR-20a-3p | 0.346246466 |
| mmu-miR-136-5p | 0.345653648 |
| mmu-miR-93-5p | 0.345028489 |
| mmu-miR-501-3p | 0.339017162 |
| mmu-miR-185-5p | 0.334268388 |
| mmu-miR-181c-3p | 0.33018161 |
| mmu-miR-132-5p | 0.313004195 |
| mmu-let-7b-3p | 0.269817823 |
| mmu-miR-501-5p | 0.266809594 |
| mmu-miR-16-1-3p | 0.26137807 |
| mmu-miR-18a-3p | 0.237932091 |
| mmu-miR-3063-5p | 0.187811383 |
| mmu-miR-92a-1-5p | 0.17040709 |
